# Supplementary material for: Persistent Southern Tomato Virus (STV) Interacts with Cucumber Mosaic and/or Pepino Mosaic Virus in Mixed- Infections Modifying Plant Symptoms, Viral Titer and Small RNA Accumulation
Source: Microorganisms. 2021 Mar 26;9(4):689. doi: 10.3390/microorganisms9040689 (PMC8066132; doi:10.3390/microorganisms9040689)
Supplement: Supplementary file 1 [file microorganisms-09-00689-s001.zip › Supplementary Materials/Table S1.docx]

**Table S1.** Primers and probes used in this work.

| **Name** | **Assay** | **Sequence (5’-3’)** | **Genomic Region** | **References** |
| --- | --- | --- | --- | --- |
| CMV_F_rtpcr | RT-qPCR | TGA TTC AGT CAC GGA GTT CGA T | 1533-1555 (CP) | - |
| CMV_R_rtpcr | RT-qPCR | TCA AAT TTC GGC AAA GGA TTA AC | 1587-1610 (CP) | - |
| CMV probe | RT-qPCR | 6FAM-AAG CTT GTT TCG CGC ATT CAA ATT CG-TAMRA | 1559- 1585 (CP) | - |
| T7-CMV_R | Transcript elaboration | **TAA TAC GAC TCA CTA TAG TCA GGG** TCA AAT TTC GGC AAA GGA TTA AC | 1587-1610 (CP) | - |
| CMV_F | Conventional RT-PCR | CGC CTC CTC CTC CTC GGA TG | 1323-1343 (CP) | - |
| CMV_R | Conventional RT-PCR | CAT CTC TGC TAT GTT CGC GG | 1668-1687 (CP) | - |
| PepMV_F | RT-qPCR | ACT CCT AGA GCT GAC CTC AC | 5126-5145 (TGB2) | Ling et al., 2007 |
| PepMV_R | RT-qPCR | TCT CCA GCA ACA GGT TGG TA | 5232-5213 (TGB2) | Ling et al., 2007 |
| PepMV probe | RT-qPCR | 6FAM-TGT CAG CTT GCA TTT ACT TCC AAA A-TAMRA | 5180-5204 (TGB2) | Ling et al., 2007 |
| T7-PepMV_R | Transcript elaboration | **TAA TAC GAC TCA CTA TAG TCA GGG** TCT CCA GCA ACA GGT TGG TA | 5232-5213 (TGB2) | - |
| PepMV_F1 | Conventional RT-PCR | CCA AAG ATG CTG GTG CCA AAG C | 5681-5702 (TGB3) | - |
| PepMV_R1 | Conventional RT-PCR | CCA CAG GTA ATT ATA TTA CCA | 6248-6268 (TGB3) | - |
| STV_F | RT-qPCR | TGC CTC CCC AGC TGT CA | 1189-1206 (CP/RdRp) | Elvira-González et al., 2018 |
| STV_R | RT-qPCR | TGC GTT GGG ATA GAG GAG TGA | 1236-1257 (CP/RdRp) | Elvira-González et al., 2018 |
| STV probe | RT-qPCR | 6FAM-CGC AAC AGA GGT AGA GGC AGA GGC C-TAMRA | 1209-1234 (CP/RdRp) | Elvira-González et al., 2018 |
| T7-STV_R | Transcript elaboration | **TAA TAC GAC TCA CTA TAG TCA GGG** TGC GTT GGG ATA GAG GAG TGA | 1236-1257 (CP/RdRp) | Elvira-González et al., 2018 |
