# Supplementary material for: Persistent Southern Tomato Virus (STV) Interacts with Cucumber Mosaic and/or Pepino Mosaic Virus in Mixed- Infections Modifying Plant Symptoms, Viral Titer and Small RNA Accumulation
Source: Microorganisms. 2021 Mar 26;9(4):689. doi: 10.3390/microorganisms9040689 (PMC8066132; doi:10.3390/microorganisms9040689)
Supplement: Supplementary file 1 [file microorganisms-09-00689-s001.zip › Supplementary Materials/Table S2.docx]

**Table S2.** Total and useful reads obtained by high throughput small RNA sequencing of STV, CMV and PepMV single and mixed tomato plants. Three biological replicates were sequenced for each virus combination and for the mock-inoculated plants.

| **Samples** | **Total Reads** | **Total Useful Reads** |
| --- | --- | --- |
| Mock plant 1 | 11049447 | 7748372 |
| Mock plant 2 | 7512326 | 5140699 |
| Mock plant 3* | 4256600 | 2831539 |
| STV 1 | 3816253 | 2287884 |
| STV 2* | 10821879 | 7447223 |
| STV 3 | 7843693 | 5429672 |
| CMV 1 | 5189500 | 2001513 |
| CMV 2 | 6990514 | 2925636 |
| CMV 3* | 7190131 | 2195301 |
| PepMV 1 | 11402409 | 4573369 |
| PepMV 2* | 14378959 | 4754936 |
| PepMV 3 | 10289785 | 3818637 |
| STV + CMV 1 | 9929524 | 4560116 |
| STV + CMV 2 | 8479770 | 3300271 |
| STV + CMV 3* | 10460441 | 2982811 |
| STV + PepMV 1* | 8454639 | 3167202 |
| STV + PepMV 2 | 8118009 | 2858518 |
| STV + PepMV 3 | 8058229 | 2769603 |
| CMV + PepMV 1 | 13536163 | 4702048 |
| CMV + PepMV 2* | 10052688 | 1829211 |
| CMV + PepMV 3 | 18927187 | 6740106 |
| STV + CMV + PepMV 1 | 15292678 | 7367472 |
| STV + CMV + PepMV 2 | 11748747 | 5624718 |
| STV + CMV + PepMV 3 | 12804145 | 6063582 |

* Biological replicates considered as outlayer by PCA analysis.
