# Supplementary material for: Persistent Southern Tomato Virus (STV) Interacts with Cucumber Mosaic and/or Pepino Mosaic Virus in Mixed- Infections Modifying Plant Symptoms, Viral Titer and Small RNA Accumulation
Source: Microorganisms. 2021 Mar 26;9(4):689. doi: 10.3390/microorganisms9040689 (PMC8066132; doi:10.3390/microorganisms9040689)
Supplement: Supplementary file 1 [file microorganisms-09-00689-s001.zip › Supplementary Materials/Table S3.docx]

**Table S3.** miRNA expressed differentially in tomato plants infected with CMV- single and STV + CMV co- infected tomato plants respect to the control mock-inoculated ones (FDR < 0.05 and for log2FC > 0.56). Differences of miRNA accumulation with values of log2FC > 0.56 were considered as significant (*). Potential functions of some miRNAs were described previously in the bibliography or determined by the psRNAtarget software whereas for other miRNAs it were not found (-).

|  | **miRNA Accumulation (Log2FC)** | |  |
| --- | --- | --- | --- |
| **Name** | **CMV** | **STV + CMV** | **Function** |
| **stu-miR393-3p** | 5.79 | 5.62 | Abiotic stress and development (Ding et al., 2017; Li et al., 2019; Zhang et al., 2020) |
| **sly-miR168a-3p** | 4.00 | 3.66 | Abiotic stress and defence against pathogens (M. Liu et al., 2018; Pentimone et al., 2018; Tripathi et al., 2018) |
| **stu-miR408b-5p** | 7.03 | 6.72 | Defence against pathogens (Stare et al., 2019) |
| **sly-miR9470-5p*** | 4.57 | 3.41 | Abiotic stress and defence against pathogens (Prigigallo et al., 2019; Zhao et al., 2017) |
| **stu-miR8031*** | -3.19 | -0.97 | - |
| **sly-miR403-5p** | 2.21 | 2.64 | Abiotic stress and defence against pathogens (Liu et al., 2017; Prigigallo et al., 2019) |
| **stu-miR167b-3p** | 4.48 | 4.40 | - |
| **sly-miR9479-3p*** | 3.96 | 2.62 | - |
| **sly-miR9475-5p** | -1.88 | -1.68 | - |
| **sly-miR169e-3p*** | -5.69 | -5.08 | Abiotic stress and defence against pathogens (Liu et al., 2017; M. Liu et al., 2018; Tripathi et al., 2018; Zhao et al., 2017) |
| **sly-miR156e-5p** | -2.62 | -2.26 | Abiotic stress (Dong et al., 2020; Kataria and Verma, 2018; M. Liu et al., 2018; Zhao et al., 2017) |
| **gma-miR6300** | 3.44 | 3.15 | - |
| **sly-miR9476-3p** | 1.65 | 1.29 | Abiotic stress, response against pathogens, electron transport and cell signalling (Filiz et al., 2019; Prigigallo et al., 2019) |
| **sly-miR9474-5p*** | -4.91 | -3.30 | Abiotic stress (Dong et al., 2020; Liu et al., 2017; M. Liu et al., 2018; Pentimone et al., 2018; Zhao et al., 2017) |
| **mtr-miR166b** | -2.56 | -2.87 | - |
| **stu-miR6024-5p*** | 4.10 | 5.04 | Resistance against pathogens (Wei et al., 2014) |
| **bta-miR-2478*** | 1.97 | 1.28 | - |
| **gma-miR396e*** | -3.17 | -1.76 | - |
| **zma-miR166i-5p** | 4.19 | No differential expression | Defence against pathogens (Yadav et al., 2015) |
| **sly-miR167b-5p** | -2.04 | -1.58 | Abiotic stress (Rey-Burusco et al., 2019) |
| **stu-miR7988*** | 3.44 | 3.27 | - |
| **mtr-miR172c-5p*** | 3.77 | 4.73 | Abiotic stress (Long et al., 2020) |
| **sly-miR9471b-3p** | -1.36 | -1.25 | Abiotic stress and defence against pathogens (M. Liu et al., 2018; Tripathi et al., 2018; Zhao et al., 2017) |
| **sly-miR9476-5p** | 1.32 | 1.11 | Defence against pathogens (Tripathi et al., 2018) |
| **sly-miR477-3p** | -3.44 | -3.63 | Abiotic stress (Filiz et al., 2019; M. Liu et al., 2018; Pentimone et al., 2018; Tripathi et al., 2018; Zhao et al., 2017) |
| **ath-miR165a-5p*** | 3.27 | 2.40 | Abiotic stress (Yang et al., 2019) |
| **stu-miR6026-5p** | 2.01 | 2.38 | - |
| **stu-miR7983-5p** | -1.99 | No differential expression | Abiotic stress and development (Kondhare et al., 2018) |
| **stu-miR482e-5p** | 2.27 | No differential expression | Defence against pathogens (Chi et al., 2015) |
| **stu-miR398a-5p** | 2.98 | 3.23 | Defence against pathogens (Stare et al., 2019; Travezaño and Patricia, 2016) |
| **bdi-miR7782-3p** | -1.78 | -1.72 | - |
| **sly-miR9478-5p** | 2.71 | 2.43 | Abiotic stress and defence against pathogens (M. Liu et al., 2018; Tripathi et al., 2018; Zhao et al., 2017) |
| **ppe-miR858** | -1.37 | No differential expression | - |
| **stu-miR398a-3p** | -1.82 | -1.97 | Abiotic stress and protection against oxidative stress (Qiao et al., 2017; Sarkar et al., 2017; Shin et al., 2017) |
| **sly-miR166c-5p** | No differential expression | -3.23 | Abiotic stress and defence against pathogens (Liu et al., 2017; M. Liu et al., 2018; Tripathi et al., 2018; Zhao et al., 2017) |
| **sly-miR164b-3p** | No differential expression | -4.00 | Abiotic stress and development (Liu et al., 2017; Yin et al., 2018; Zhao et al., 2017) |
| **sly-miR482e-5p** | No differential expression | -2.08 | Abiotic stress and defence against pathogens (Liu et al., 2017; Tripathi et al., 2018; Zhao et al., 2017) |
| **sly-miR9470-3p** | No differential expression | -1.55 | Defence against pathogens (Pentimone et al., 2018) |
| **ppe-miR396a** | No differential expression | -3.69 | Development (Farinati et al., 2020) |
| **sly-miR9473-5p** | No differential expression | 1.76 | Defence against pathogens (Pentimone et al., 2018) |
| **mmu-miR-466i-5p** | No differential expression | 4.06 | Development (psRNAtarget software) |
| **stu-miR156d-3p** | No differential expression | -2.11 | Abiotic stress (Shin et al., 2017) |
| **stu-miR8025-3p** | No differential expression | 3.59 | - |
| **stu-miR319-3p** | No differential expression | -2.01 | Plant development (Chaves et al., 2015; Kondhare et al., 2018) |
| **stu-miR396-3p** | No differential expression | 1.12 | - |
| **stu-miR3627-5p** | No differential expression | 3.61 | - |
| **osa-miR162b** | No differential expression | -1.97 | Abiotic stress (Goswami et al., 2017; Li et al., 2015) |
| **stu-miR167d-3p** | No differential expression | -1.13 | Abiotic stress and development (Zhang et al., 2019) |
| **gma-miR172k** | No differential expression | 2.42 | Abiotic stress and development (Akdogan et al., 2016; C. Liu et al., 2018; Wang et al., 2016; Wu et al., 2017) |
| **mdm-miR396a** | No differential expression | -1.53 | Development (Farinati et al., 2020) |
| **bdi-miR845** | No differential expression |  | Multifunction (DNA repair and transcription regulation) (W. Liu et al., 2018) |
| **sly-miR482d-5p** | No differential expression | -2.03 | Defence against pathogens (Tripathi et al., 2018) |
| **sly-miR403-3p** | No differential expression | 0.79 | - |
| **sly-miR1916** | No differential expression | -2.30 | Abiotic stress, development and pathogens (Chen et al., 2019b, 2019a; Feng et al., 2014; Mohorianu et al., 2011; Moxon et al., 2008; Pentimone et al., 2018) |
| **ptc-miR6478** | No differential expression | -3.13 | Abiotic stress and development (He et al., 2015; Zeng et al., 2019; Żywicki et al., 2015) |
| **sly-miR4376** | No differential expression | 1.21 | Defence against pathogens (Feng et al., 2014; Pradhan et al., 2015; Tripathi et al., 2018) |
| **sly-miR9471b-5p** | No differential expression | 1.67 | Abiotic stress and defence against pathogens (Liu et al., 2017; Tripathi et al., 2018) |
| **osa-miR166g-5p** | No differential expression | -1.62 | - |
| **sly-miR9472-5p** | No differential expression | -1.55 | Abiotic stress, defence against pathogens (M. Liu et al., 2018; Tripathi et al., 2018) |
| **sly-miR164a-3p** | No differential expression | -1.02 | Abiotic stress (Liu et al., 2017; M. Liu et al., 2018) |
| **ath-miR8175** | No differential expression | 1.10 | Abiotic stress (Wu et al., 2018; Zeng et al., 2019) |
| **sly-miR164a-3p** | No differential expression | -1.02 | Abiotic stress (Liu et al., 2017; M. Liu et al., 2018) |
