# Supplementary material for: Persistent Southern Tomato Virus (STV) Interacts with Cucumber Mosaic and/or Pepino Mosaic Virus in Mixed- Infections Modifying Plant Symptoms, Viral Titer and Small RNA Accumulation
Source: Microorganisms. 2021 Mar 26;9(4):689. doi: 10.3390/microorganisms9040689 (PMC8066132; doi:10.3390/microorganisms9040689)
Supplement: Supplementary file 1 [file microorganisms-09-00689-s001.zip › Supplementary Materials/Table S4.docx]

**Table S4.** miRNA expressed differentially in tomato plants infected with PepMV- single and STV + PepMV co- infected tomato plants respect to the control mock-inoculated ones (FDR < 0.05 and for log2FC > 0.56). Differences of miRNA accumulation with values of log2FC > 0.56 were considered as significant (*). Potential functions of some miRNAs were described previously in the bibliography or determined by the psRNAtarget software whereas for other miRNAs it were not found (-).

|  | **miRNA Accumulation (Log2FC)** | |  |
| --- | --- | --- | --- |
| **Name** | **PepMV** | **STV + PepMV** | **Function** |
| **stu-miR398a-5p** | No differential expression | 3.46 | Defence against pathogens (Stare et al., 2019; Travezaño and Patricia, 2016) |
| **sly-miR169e-3p** | No differential expression | -2.68 | Abiotic stress and defence against pathogens (Liu et al., 2017; M. Liu et al., 2018; Tripathi et al., 2018; Zhao et al., 2017) |
| **stu-miR319-3p** | -1.72 | -1.92 | Plant development (Chaves et al., 2015; Kondhare et al., 2018) |
| **stu-miR393-3p** | 1.47 | No differential expression | Abiotic stress and development (Ding et al., 2017; Li et al., 2019; Zhang et al., 2020) |
| **stu-miR408b-5p** | 3.00 | 3.32 | Defence against pathogens (Stare et al., 2019) |
| **sly-miR9470-5p*** | 4.89 | 5.73 | Abiotic stress and defence against pathogens (Prigigallo et al., 2019; Zhao et al., 2017) |
| **stu-miR8031** | -0.97 | No differential expression | - |
| **sly-miR9479-3p** | 2.13 | 2.36 | - |
| **sly-miR9475-5p** | -2.01 | -2.09 | - |
| **sly-miR156e-5p*** | -2.55 | -1.55 | Abiotic stress (Dong et al., 2020; Kataria and Verma, 2018; M. Liu et al., 2018; Zhao et al., 2017) |
| **gma-miR6300** | 2.78 | 3.30 | - |
| **sly-miR9476-3p** | 1.87 | 1.74 | Abiotic stress, response against pathogens, electron transport and cell signalling (Filiz et al., 2019; Prigigallo et al., 2019) |
| **sly-miR9474-5p*** | -2.58 | -5.17 | Abiotic stress (Dong et al., 2020; Liu et al., 2017; M. Liu et al., 2018; Pentimone et al., 2018; Zhao et al., 2017) |
| **mtr-miR166b** | -1.39 | -1.54 | - |
| **stu-miR6024-5p*** | 2.71 | 1.42 | Resistance against pathogens (Wei et al., 2014) |
|  |  |  |  |
| **bta-miR-2478*** | 1.98 | 2.56 | - |
| **gma-miR396e** | -2.10 | -1.97 | - |
| **sly-miR167b-5p** | -1.80 | -1.72 | Abiotic stress (Rey-Burusco et al., 2019) |
| **sly-miR9471b-3p** | -1.68 | -1.47 | Abiotic stress and defence against pathogens (M. Liu et al., 2018; Tripathi et al., 2018; Zhao et al., 2017) |
| **sly-miR477-3p** | -1.94 | No differential expression | Abiotic stress (Filiz et al., 2019; M. Liu et al., 2018; Pentimone et al., 2018; Tripathi et al., 2018; Zhao et al., 2017) |
| **bdi-miR7782-3p** | -1.35 | No differential expression | - |
| **stu-miR167d-3p** | 1.71 | 1.30 | Abiotic stress and development (Zhang et al., 2019) |
| **ptc-miR6478** | 1.33 | 1.77 | Abiotic stress and development (He et al., 2015; Zeng et al., 2019; Żywicki et al., 2015) |
| **stu-miR396-3p** | 1.40 | 1.00 | - |
| **sly-miR164b-3p** | -2.13 | -2.59 | Abiotic stress and development (Liu et al., 2017; Yin et al., 2018; Zhao et al., 2017) |
| **osa-miR162b** | -3.13 | -2.82 | Abiotic stress (Goswami et al., 2017; Li et al., 2015) |
| **stu-miR166d-5p** | 2.51 | 2.32 | Abiotic stress (Deng et al., 2018) |
| **ppe-miR396a** | -2.33 | -2.91 | Development (Farinati et al., 2020) |
| **stu-miR167c-3p** | 1.62 | No differential expression | - |
| **sly-miR482e-5p** | 1.07 | No differential expression | Abiotic stress and defence against pathogens (Liu et al., 2017; Tripathi et al., 2018; Zhao et al., 2017) |
| **stu-miR156d-3p** | -1.46 | -1.33 | Abiotic stress (Shin et al., 2017) |
| **sly-miR5300** | 0.98 | No differential expression | Abiotic stress and defence against pathogens (Pentimone et al., 2018; Tripathi et al., 2018; Zhao et al., 2017) |
| **stu-miR7980b-3p** | 1.53 | 1.66 | - |
| **ath-miR8175** | 1.43 | 1.86 | Abiotic stress (Wu et al., 2018; Zeng et al., 2019) |
| **sly-miR6024** | -0.92 | -1.33 | Defence against pathogens (Filiz et al., 2019; Niu et al., 2015; Wei et al., 2014) |
| **bdi-miR845** | -2.40 | No differential expression | Multifunction (DNA repair and transcription regulation) (W. Liu et al., 2018) |
| **bdi-miR162** | -1.89 | No differential expression | - |
| **sly-miR9471a-3p** | -0.77 | No differential expression | Abiotic stress and defence against pathogens (M. Liu et al., 2018; Pentimone et al., 2018; Zhao et al., 2017) |
| **mmu-miR-8117*** | 1.15 | 2.32 | - |
| **zma-miR396g-3p** | 2.21 | No differential expression | - |
| **osa-miR1873** | -2.29 | No differential expression | Defence against pathogens (Zhou et al., 2019) |
| **sly-miR9470-3p** | No differential expression | 1.13 | Defence against pathogens (Pentimone et al., 2018) |
| **stu-miR3627-5p** | No differential expression | 3.27 | - |
| **sly-miR403-3p** | No differential expression | -0.93 | - |
| **sly-miR1916** | No differential expression | -2.38 | Abiotic stress, development and pathogens (Chen et al., 2019b, 2019a; Feng et al., 2014; Mohorianu et al., 2011; Moxon et al., 2008; Pentimone et al., 2018) |
| **sly-miR164a-3p** | No differential expression | -1.29 | Abiotic stress (Liu et al., 2017; M. Liu et al., 2018) |
| **ppt-miR894** | No differential expression | 1.35 | Abiotic stress and development (Kantar et al., 2011; Li et al., 2009; Wei et al., 2009) |
| **osa-miR5072** | No differential expression | 2.02 | Abiotic stress and defence against pathogens (Dubey et al., 2020; Maeda et al., 2016; Xu et al., 2014) |
| **vvi-miR3630-3p** | No differential expression | 1.94 | - |
