# Supplementary material for: Persistent Southern Tomato Virus (STV) Interacts with Cucumber Mosaic and/or Pepino Mosaic Virus in Mixed- Infections Modifying Plant Symptoms, Viral Titer and Small RNA Accumulation
Source: Microorganisms. 2021 Mar 26;9(4):689. doi: 10.3390/microorganisms9040689 (PMC8066132; doi:10.3390/microorganisms9040689)
Supplement: Supplementary file 1 [file microorganisms-09-00689-s001.zip › Supplementary Materials/Table S5.docx]

**Table S5.** miRNA expressed differentially in tomato plants infected with PepMV or CMV- single and CMV + PepMV co- infected tomato plants respect to the control mock-inoculated ones (FDR < 0.05 and for log2FC > 0.56). Differences of miRNA accumulation with values of log2FC > 0.56 were considered as significant (*). Potential functions of some miRNAs were described previously in the bibliography or determined by the psRNAtarget software whereas for other miRNAs, it were not found (-).

|  | **miRNA Accumulation (Log2FC)** | | |  |
| --- | --- | --- | --- | --- |
| **Name** | **CMV** | **PepMV** | **CMV + PepMV** | **Function** |
| **stu-miR393-3p*** | 5.79 | 1.47 | 2.18 | Abiotic stress and development (Ding et al., 2017; Li et al., 2019; Zhang et al., 2020) |
| **sly-miR168a-3p** | 4.00 | No differential expression | No differential expression | Abiotic stress and defence against pathogens (M. Liu et al., 2018; Pentimone et al., 2018; Tripathi et al., 2018) |
| **stu-miR408b-5p*** | 7.03 | 3.00 | No differential expression | Defence against pathogens (Stare et al., 2019) |
| **sly-miR9470-5p*** | 4.57 | 4.89 | 5.75 | Abiotic stress and defence against pathogens (Prigigallo et al., 2019; Zhao et al., 2017) |
| **stu-miR8031*** | -3.19 | -0.97 | No differential expression | - |
| **sly-miR403-5p** | 2.21 | No differential expression | No differential expression | Abiotic stress and defence against pathogens (Liu et al., 2017; Prigigallo et al., 2019) |
| **stu-miR167b-3p** | 4.48 | No differential expression | No differential expression | - |
| **sly-miR9479-3p*** | 3.96 | 2.13 | No differential expression | - |
| **sly-miR9475-5p*** | -1.88 | -2.01 | -2.90 | - |
| **sly-miR169e-3p*** | -5.69 | No differential expression | -3.64 | Abiotic stress and defence against pathogens (Liu et al., 2017; M. Liu et al., 2018; Tripathi et al., 2018; Zhao et al., 2017) |
| **sly-miR156e-5p** | -2.62 | -2.55 | -2.03 | Abiotic stress (Dong et al., 2020; Kataria and Verma, 2018; M. Liu et al., 2018; Zhao et al., 2017) |
| **gma-miR6300*** | 3.44 | 2.78 | 3.63 | - |
| **sly-miR9476-3p** | 1.65 | 1.87 | No differential expression | Abiotic stress, response against pathogens, electron transport and cell signalling (Filiz et al., 2019; Prigigallo et al., 2019) |
| **sly-miR9474-5p*** | -4.91 | -2.58 | -3.72 | Abiotic stress (Dong et al., 2020; Liu et al., 2017; M. Liu et al., 2018; Pentimone et al., 2018; Zhao et al., 2017) |
| **mtr-miR166b*** | -2.56 | -1.39 | No differential expression | - |
| **stu-miR6024-5p*** | 4.10 | 2.71 | No differential expression | Resistance against pathogens (Wei et al., 2014) |
| **bta-miR-2478** | 1.97 | 1.98 | 1.84 | - |
| **gma-miR396e*** | -3.17 | -2.10 | -1.63 | - |
| **zma-miR166i-5p** | 4.19 | No differential expression | No differential expression | Defence against pathogens (Yadav et al., 2015) |
| **sly-miR167b-5p** | -2.04 | -1.80 | -1.78 | Abiotic stress (Rey-Burusco et al., 2019) |
| **stu-miR7988** | 3.44 | No differential expression | No differential expression | - |
| **mtr-miR172c-5p** | 3.77 | No differential expression | No differential expression | Abiotic stress (Long et al., 2020) |
| **sly-miR9471b-3p*** | -1.36 | -1.68 | -1.10 | Abiotic stress and defence against pathogens (M. Liu et al., 2018; Tripathi et al., 2018; Zhao et al., 2017) |
| **sly-miR9476-5p** | 1.32 | No differential expression | No differential expression | Defence against pathogens (Tripathi et al., 2018) |
| **sly-miR477-3p*** | -3.44 | -1.94 | -2.24 | Abiotic stress (Filiz et al., 2019; M. Liu et al., 2018; Pentimone et al., 2018; Tripathi et al., 2018; Zhao et al., 2017) |
| **ath-miR165a-5p** | 3.27 | No differential expression | No differential expression | Abiotic stress (Yang et al., 2019) |
| **stu-miR6026-5p** | 2.01 | No differential expression | No differential expression | - |
| **stu-miR7983-5p** | -1.99 | No differential expression | No differential expression | Abiotic stress and development (Kondhare et al., 2018) |
| **stu-miR482e-5p** | 2.27 | No differential expression | No differential expression | Defence against pathogens (Chi et al., 2015) |
| **stu-miR398a-5p** | 2.98 | No differential expression | No differential expression | Defence against pathogens (Stare et al., 2019; Travezaño and Patricia, 2016) |
| **bdi-miR7782-3p** | -1.78 | -1.35 | No differential expression | - |
| **sly-miR9478-5p** | 2.71 | No differential expression | No differential expression | Abiotic stress and defence against pathogens (M. Liu et al., 2018; Tripathi et al., 2018; Zhao et al., 2017) |
| **ppe-miR858** | -1.37 | No differential expression | No differential expression | - |
| **stu-miR398a-3p** | -1.82 | No differential expression | -2.10 | Abiotic stress and protection against oxidative stress (Qiao et al., 2017; Sarkar et al., 2017; Shin et al., 2017) |
| **stu-miR319-3p*** | No differential expression | -1.72 | -2.59 | Plant development (Chaves et al., 2015; Kondhare et al., 2018) |
| **stu-miR167d-3p** | No differential expression | 1.71 | 1.41 | Abiotic stress and development (Zhang et al., 2019) |
| **ptc-miR6478** | No differential expression | 1.33 | No differential expression | Abiotic stress and development (He et al., 2015; Zeng et al., 2019; Żywicki et al., 2015) |
| **stu-miR396-3p*** | No differential expression | 1.40 | 0.87 | - |
| **sly-miR164b-3p** | No differential expression | -2.13 | No differential expression | Abiotic stress and development (Liu et al., 2017; Yin et al., 2018; Zhao et al., 2017) |
| **osa-miR162b** | No differential expression | -3.13 | No differential expression | Abiotic stress (Goswami et al., 2017; Li et al., 2015) |
| **stu-miR166d-5p** | No differential expression | 2.51 | No differential expression | Abiotic stress (Deng et al., 2018) |
| **ppe-miR396a** | No differential expression | -2.33 | No differential expression | Development (Farinati et al., 2020) |
| **stu-miR167c-3p** | No differential expression | 1.62 | No differential expression | - |
| **sly-miR482e-5p** | No differential expression | 1.07 | No differential expression | Abiotic stress and defence against pathogens (Liu et al., 2017; Tripathi et al., 2018; Zhao et al., 2017) |
| **stu-miR156d-3p** | No differential expression | -1.46 | No differential expression | Abiotic stress (Shin et al., 2017) |
| **sly-miR5300** | No differential expression | 0.98 | No differential expression | Abiotic stress and defence against pathogens (Pentimone et al., 2018; Tripathi et al., 2018; Zhao et al., 2017) |
| **stu-miR7980b-3p** | No differential expression | 1.53 | 1.64 | - |
| **ath-miR8175** | No differential expression | 1.43 | 1.23 | Abiotic stress (Wu et al., 2018; Zeng et al., 2019) |
| **sly-miR6024** | No differential expression | -0.92 | -1.14 | Defence against pathogens (Filiz et al., 2019; Niu et al., 2015; Wei et al., 2014) |
| **bdi-miR845** | No differential expression | -2.40 | No differential expression | Multifunction (DNA repair and transcription regulation) (W. Liu et al., 2018) |
| **bdi-miR162** | No differential expression | -1.89 | -1.94 | - |
| **sly-miR9471a-3p** | No differential expression | -0.77 | -1.06 | Abiotic stress and defence against pathogens (M. Liu et al., 2018; Pentimone et al., 2018; Zhao et al., 2017) |
| **mmu-miR-8117** | No differential expression | 1.15 | No differential expression | - |
| **zma-miR396g-3p** | No differential expression | 2.21 | No differential expression | - |
| **osa-miR1873** | No differential expression | -2.29 | No differential expression | Defence against pathogens (Zhou et al., 2019) |
| **stu-miR3627-5p** | No differential expression | No differential expression | 4.06 | - |
| **ppt-miR894** | No differential expression | No differential expression | 1.34 | Abiotic stress and development (Kantar et al., 2011; Li et al., 2009; Wei et al., 2009) |
| **sly-miR9475-3p** | No differential expression | No differential expression | -2.76 | - |
