# Supplementary material for: Persistent Southern Tomato Virus (STV) Interacts with Cucumber Mosaic and/or Pepino Mosaic Virus in Mixed- Infections Modifying Plant Symptoms, Viral Titer and Small RNA Accumulation
Source: Microorganisms. 2021 Mar 26;9(4):689. doi: 10.3390/microorganisms9040689 (PMC8066132; doi:10.3390/microorganisms9040689)
Supplement: Supplementary file 1 [file microorganisms-09-00689-s001.zip › Supplementary Materials/Table S6.docx]

**Table S6.** miRNA expressed differentially in tomato plants infected with CMV + PepMV co- infected and STV + CMV + PepMV triple- infected tomato plants respect to the control mock-inoculated ones (FDR < 0.05 and for log2FC > 0.56). Differences of miRNA accumulation with values of log2FC > 0.56 were considered as significant (*). Potential functions of some miRNAs were described previously in the bibliography or determined by the psRNAtarget software whereas for other miRNAs it were not found (-).

|  | **miRNA Accumulation (Log2FC)** | |  |
| --- | --- | --- | --- |
| **Name** | **CMV + PepMV** | **STV + CMV + PepMV** | **Function** |
| **stu-miR398a-3p** | -2.10 | No differential expression | Abiotic stress and protection against oxidative stress (Qiao et al., 2017; Sarkar et al., 2017; Shin et al., 2017) |
| **stu-miR398a-5p** | No differential expression | -2.98 | Defence against pathogens (Stare et al., 2019; Travezaño and Patricia, 2016) |
| **sly-miR169e-3p** | -3.64 | No differential expression | Abiotic stress and defence against pathogens (Liu et al., 2017; M. Liu et al., 2018; Tripathi et al., 2018; Zhao et al., 2017) |
| **stu-miR319-3p** | -2.59 | No differential expression | Plant development (Chaves et al., 2015; Kondhare et al., 2018) |
| **stu-miR393-3p*** | 2.18 | -3.84 | Abiotic stress and development (Ding et al., 2017; Li et al., 2019; Zhang et al., 2020) |
| **sly-miR168a-3p** | No differential expression | -3.04 | Abiotic stress and defence against pathogens (M. Liu et al., 2018; Pentimone et al., 2018; Tripathi et al., 2018) |
| **stu-miR408b-5p** | No differential expression | -6.11 | Defence against pathogens (Stare et al., 2019) |
| **sly-miR9470-5p*** | 5.75 | 0.57 | Abiotic stress and defence against pathogens (Prigigallo et al., 2019; Zhao et al., 2017) |
| **stu-miR8031** | No differential expression | 1.31 | - |
| **sly-miR403-5p** | No differential expression | -2.73 | Abiotic stress and defence against pathogens (Liu et al., 2017; Prigigallo et al., 2019) |
| **stu-miR167b-3p** | No differential expression | -2.47 | - |
| **sly-miR9475-5p** | -2.90 | No differential expression | - |
| **sly-miR156e-5p** | -2.03 | No differential expression | Abiotic stress (Dong et al., 2020; Kataria and Verma, 2018; M. Liu et al., 2018; Zhao et al., 2017) |
| **gma-miR6300** | 3.63 | No differential expression | - |
| **sly-miR9474-5p** | -3.72 | No differential expression | Abiotic stress (Dong et al., 2020; Liu et al., 2017; M. Liu et al., 2018; Pentimone et al., 2018; Zhao et al., 2017) |
| **mtr-miR166b** | No differential expression | 1.09 | - |
| **stu-miR6024-5p** | No differential expression | -3.80 | Resistance against pathogens (Wei et al., 2014) |
| **bta-miR-2478** | 1.84 | 1.33 | - |
| **gma-miR396e** | -1.63 | No differential expression | - |
| **sly-miR167b-5p** | -1.78 | No differential expression | Abiotic stress (Rey-Burusco et al., 2019) |
| **mtr-miR172c-5p** | No differential expression | -2.75 | Abiotic stress (Long et al., 2020) |
| **sly-miR9471b-3p** | -1.10 | No differential expression | Abiotic stress and defence against pathogens (M. Liu et al., 2018; Tripathi et al., 2018; Zhao et al., 2017) |
| **sly-miR477-3p** | -2.24 | No differential expression | Abiotic stress (Filiz et al., 2019; M. Liu et al., 2018; Pentimone et al., 2018; Tripathi et al., 2018; Zhao et al., 2017) |
| **stu-miR6026-5p** | No differential expression | -2.45 | - |
| **stu-miR167d-3p** | 1.41 | - | Abiotic stress and development (Zhang et al., 2019) |
| **ptc-miR6478** | No differential expression | 0.62 | Abiotic stress and development (He et al., 2015; Zeng et al., 2019; Żywicki et al., 2015) |
| **stu-miR396-3p** | 0.87 | No differential expression | - |
| **stu-miR7980b-3p** | 1.64 | No differential expression | - |
| **ath-miR8175** | 1.23 | 1.09 | Abiotic stress (Wu et al., 2018; Zeng et al., 2019) |
| **sly-miR6024** | -1.14 | No differential expression | Defence against pathogens (Filiz et al., 2019; Niu et al., 2015; Wei et al., 2014) |
| **bdi-miR162** | -1.94 | No differential expression | - |
| **sly-miR9471a-3p** | -1.06 | -0.79 | Abiotic stress and defence against pathogens (M. Liu et al., 2018; Pentimone et al., 2018; Zhao et al., 2017) |
| **sly-miR166c-5p** | No differential expression | 1.42 | Abiotic stress and defence against pathogens (Liu et al., 2017; M. Liu et al., 2018; Tripathi et al., 2018; Zhao et al., 2017) |
| **sly-miR9470-3p** | No differential expression | 1.08 | Defence against pathogens (Pentimone et al., 2018) |
| **sly-miR9473-5p** | No differential expression | -1.42 | Defence against pathogens (Pentimone et al., 2018) |
| **stu-miR3627-5p** | 4.06 | No differential expression | - |
| **mdm-miR396a** | No differential expression | 1.58 | Development (Farinati et al., 2020) |
| **sly-miR403-3p** | No differential expression | -1.68 | - |
| **sly-miR4376** | No differential expression | -1.17 | Defence against pathogens (Feng et al., 2014; Pradhan et al., 2015; Tripathi et al., 2018) |
| **ppt-miR894** | 1.34 | 1.16 | Abiotic stress and development (Kantar et al., 2011; Li et al., 2009; Wei et al., 2009) |
| **sly-miR9475-3p** | -2.76 | No differential expression | - |
| **stu-miR4376-5p** | No differential expression | -1.04 | Abiotic stress and defence against pathogens (Rey-Burusco et al., 2019; Stare et al., 2019) |
| **sly-miR167b-3p** | No differential expression | -0.84 | Abiotic stress and defence against pathogens (Feng et al., 2014; Liu et al., 2017; M. Liu et al., 2018; Tripathi et al., 2018; Zhao et al., 2017) |
